# Doppler ultrasonography reveals blood flow signals within the masses of invasive moles in subjects with normal hCG levels after chemotherapy: Three case reports

**DOI:** 10.3892/ol.2013.1474

**Published:** 2013-07-17

**Authors:** XI ZHOU, ZHAO DUAN

**Affiliations:** 1 Reproductive Research Center, Renmin Hospital, Hubei University of Medicine, Shiyan, Hubei 442000, P.R. China; 2 Department of Radiation Oncology, The First Affiliated Hospital of the Medical College, Xi’an Jiaotong University, Xi’an, Shaanxi 710004, P.R. China; 3 Department of Obstetrics and Gynecology, The Second Affiliated Hospital of The Medical College, Xi’an Jiaotong University, Xi’an, Shaanxi 710061, P.R. China

**Keywords:** Doppler ultrasonography, blood-flow signal, chemotherapy, invasive moles, human chorionic gonadotrophin

## Abstract

A consensus has formed that patients with invasive moles should continue with one to three cycles of chemotherapy after human chorionic gonadotropin (hCG) levels have decreased to a normal level. However, the management plan has not been agreed for cases where Doppler ultrasonography (DU) indicates blood-flow signals within the tumor mass after one to three cycles of chemotherapy when the hCG concentration has returned to normal. The present study describes the clinical and therapeutic courses of three patients with invasive moles with confirmed blood-flow signals (by DU) after their hCG levels had normalized. One patient underwent surgery to remove the mass within the uterine muscle, while the other two patients decided to cease chemotherapy and were managed by follow-up appointments; all three patients had good outcomes. These data illustrate that patients with invasive moles should be followed up if DU indicates blood-flow signals in the tumor mass after one to three cycles of chemotherapy when the hCG level has decreased back to a normal level.

## Introduction

Gestational trophoblastic disease (GTD) is known to be associated with increased maternal age and is more commonly observed in Asian subjects compared with non-Asian subjects (1). GTD may be subcategorized into hydatidiform moles [complete hydatidiform moles (CHMs), partial hydatidiform moles (PHMs) and invasive moles (IMs)] and gestational trophoblastic tumors [gestational choriocarcinomas (GTTs), placental site trophoblastic tumors (PSTT) and epithelioid trophoblastic tumors (ETTs)] (1)

After malignant transformation of the mole, chemotherapy is indicated. IMs are divided into low-risk or high-risk based on each prognostic factor, including age, human chorionic gonadotropin (hCG) levels, antecedent pregnancy, interval from antecedent pregnancy to chemotherapy, metastases, largest tumor mass diameter and previous chemotherapy. This was intensively revised by Seckl *et al*(1) and Tse *et al*(2) that how to estimate the IM risk. In cases of low-risk disease (score 0–6), therapy is continued for six weeks (one to two cycles of chemotherapy). In cases of high-risk disease (score ≥7), therapy lasts for eight weeks (two to three cycles of chemotherapy) if poor prognostic features such as metastases to the liver or brain are present (1). However, in a small number of patients with IMs, Doppler ultrasonography (DU) indicates blood-flow signals within the mass if one to two cycles of chemotherapy have been completed after their hCG levels have decreased back to normal. The dilemma is which treatment option should be pursued, a simple follow-up, continued chemotherapy or a resection of the mass. The present study describes three patients in whom DU indicated blood-flow signals in the tumor mass after one to two cycles of chemotherapy when the patients’ hCG concentrations had returned to a normal level. This study was approved by the Medical Ethics Committee of Hubei University of Medicine (Hubei, China) and the patients all provided informed written consent.

## Case reports

### Case 1

**Figure 1:**
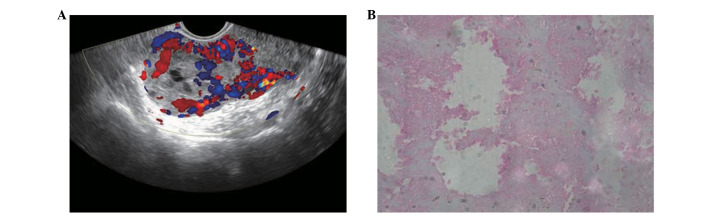
(A) Doppler ultrasonography (DU) showed rich blood-flow signals during the second cycle of chemotherapy after the human chorionic gonadotrophin (hCG) level decreased to normal levels. (B) The pathological section indicated necrosis of the chorionic villus (HE staining; magnification, ×400). HE, hematoxylin and eosin.

Case 1 was that of a 38-year-old female (gravida 4, para 1) who experienced vaginal bleeding 12 weeks into pregnancy. Transvaginal ultrasonography (TVS) indicated that the uterine cavity was filled with cystic material. The attending physician suggested that this was a molar pregnancy. Aspiration was performed. The pathological diagnosis was of a CHM. The hCG level increased from 2,130 mIU/ml to 4,321 mIU/ml (normal range, 0–5 mIU/ml) continually for three weeks after TVS. Rich blood-flow signals within the myometrium of the uterus were noted. The patient was subsequently diagnosed as having an IM (score, 5). The patient underwent five cycles of chemotherapy consisting of 5-fluorouracil (5-FU; 26 mg/kg/day, 8 days/cycle) and actinomycin (KSM; 6 μg/kg/day, 8 days/cycle); the interval between each cycle was three weeks. At the beginning of the fourth cycle, the hCG level decreased to 0.1 mIU/ml. Subsequent to this cycle, menstruation started again. At the fifth cycle, DU (5–7.5 MHz transvaginal transducer with spectral Doppler) indicated that rich blood-flow signals were present in the mass within the myometrium (Fig. 1). The resistive index (RI) was 0.24 and the number of blood-flow signals had sharply decreased compared with the start of chemotherapy. The patient requested resection of the mass. After entering the abdomen, it was observed that the mass was on the convex surface of the uterus. The color of the surface of the mass was identical to that of the normal tissue. The pathological diagnosis of the mass was necrosis of the placental tissue (Fig. 1). Another cycle of chemotherapy was administered following the surgery. The patient’s hCG level remained with the normal range during the 17 months of follow-up.

### Case 2

Case 2 was that of a 22-year-old female (gravida 2, para 0) at the 16th week of pregnancy. The patient was diagnosed as having a hydatidiform mole due to vaginal bleeding, high hCG levels and indication of a cystic pregnancy by TVS. Subsequent to aspiration, the pathological diagnosis was of a CHM. The patient’s hCG level decreased for one week, then constantly increased over the next three weeks. DU revealed rich blood-flow signals within the myometrium of the uterus. The patient was diagnosed as having an IM (score, 8) and subsequently underwent four cycles of chemotherapy with etoposide (100 mg/m^2^), methotrexate (200 mg/m^2^), KSM (6 μg/kg), cyclophosphamide (100 mg/m^2^) and vincristine (1 mg/m^2^; EMA-CO regimen). The interval between each cycle was three weeks. The hCG level decreased to within the normal range at the beginning of the third cycle. Following the fourth cycle of chemotherapy, the patient opted to be managed by follow-up appointments even though DU showed that blood-flow signals were present within the mass (RI, 0.21). The patient’s hCG level was monitored through the follow-up appointments and remained normal. Repeated DU indicated that the blood-flow signals had disappeared after six months. After one year of chemotherapy, the patient became pregnant again and the pre-natal diagnosis was normal. The patient delivered a healthy boy at 39 weeks of pregnancy and demonstrated a normal hCG level six months after the delivery.

### Case 3

Case 3 was that of a 34-year-old female (gravida 2, para 1) who was known to have pelvic inflammatory disease. The patient underwent *in vitro* fertilization-embryo transfer (IVF-ET) subsequent to being diagnosed with a blockage of the fallopian tubes. The patient experienced vaginal bleeding at 16 weeks of pregnancy. Repeat TVS revealed two viable fetuses, although one placenta was identified as cystic. After careful consideration, the patient and her partner decided to terminate the pregnancy and delivery was induced with rivanol. A histological examination confirmed the clinical impression of one normal placenta and a second PHM (69, XXY). Three weeks after curettage, the serial serum hCG concentrations increased continually and TVS indicated that the trophoblast had invaded the uterine myometrium. CT of the chest indicated that the trophoblast had metastasized to the lung (score, 8). Following three cycles of 5-FU with KSM, the hCG level decreased into the normal range. Subsequent to the next two cycles of chemotherapy, CT of the chest indicated that the metastasis had disappeared. However, TVS revealed blood-flow signals within the mass (RI, 0.31). The patient decided to temporarily stop therapy and wished to be managed by follow-up appointments. Two months after ceasing chemotherapy, DU did not reveal any blood-flow signals within the mass. The patient’s hCG level remained normal for the following 12 months.

## Discussion

The combined use of grayscale and color DU is able to depict the patency and flow dynamics of larger vessels in tumors. The analysis of spectral waveforms has met with variable success with regard to differentiating between benign and malignant lesions (3–5). A few studies have reported that a reduction in the number of blood-flow signals indicates the efficiency of tumor therapy in malignant lesions in mice (6,7). The use of contrast-enhanced ultrasound with microbubbles is capable of improving the accuracy of the diagnosis. However, limitations in spatial resolution, operator dependence, the short time window available for imaging and the limited field of view restrict the widespread use of microbubble ultrasound. Positron emission tomography (PET) with ^18^F-fluorodeoxyglucose may aid in the identification of the site of active disease to aid in a resection and cure (8,9).

An HM results in pregnancies with excessive proliferation of placental villi, but severely stunted or absent embryonic development. HM should be regarded as a pre-malignant lesion since 15–20% of CHMs and 1% of PHMs undergo malignant transformation into IMs, choriocarcinomas or, rarely, PSTTs (1). The most significant problem is that it is difficult to predict which HMs are likely to undergo malignant transformation. Patients must wait a number of weeks or months to find out if they require chemotherapy after the diagnosis of a molar pregnancy. Certain genetic disorders have been identified as being expressed in moles and they indicated the malignant transformation of HMs (10,11). However, it is unknown whether residual CHMs acquire additional genetic changes after evacuation; these mutations may promote malignant transformation. IMs are usually diagnosed clinically rather than pathologically, based on the persistent elevation of hCG levels following molar evacuation, and are frequently treated with chemotherapy without a histopathological diagnosis. Combination chemotherapy consisting of 5-FU and KSM or EMA-CO has been used as to treat gestational trophoblastic neoplasia (GTN) in China. Patients with GTN who progress during or after primary chemotherapy have excellent outcomes, with ~100% of low-risk and ~84% of high-risk patients being cured.

In the present cases, the hCG levels returned to normal and TVS indicated blood-flow signals within the masses. Subsequent to reviewing these cases, we suggest that IM patients should continue to be followed up if they complete regular chemotherapy.
